# Engaging traditional barbers to identify and refer newborns for routine immunization services in Sokoto, Nigeria: a mixed methods evaluation

**DOI:** 10.1007/s00038-020-01518-9

**Published:** 2020-11-02

**Authors:** Leanne Dougherty, Masduk Abdulkarim, Aliyu Ahmed, Yakubu Cherima, Aliyu Ladan, Sale Abdu, Bello Kilgori, Folake Olayinka, Sani Garr, Kate E. Gilroy

**Affiliations:** 1 Maternal and Child Survival Program (MCSP), John Snow, Inc. (JSI), 2733 Crystal Dr 4th Floor, Arlington, VA 22202 USA; 2 Data Research and Mapping Consult Ltd, Lagos, Nigeria

**Keywords:** Immunization, Interpersonal communication, Nigeria, Referral

## Abstract

**Objectives:**

This study evaluates the effectiveness of an intervention that engaged traditional barbers to inform parents about the importance of vaccination and then refer newborns for vaccination services.

**Methods:**

We conducted a pre-post quasi-experimental study (*n *= 2639) to evaluate changes in the coverage of three birth antigens among children aged 0–5 months in response to the intervention. We also conducted in-depth interviews and focus group discussions to assess the enabling factors and challenges associated with implementation.

**Results:**

We found mothers who received a yellow referral card from a traditional barber were two to three times more likely to vaccinate their children with the three birth antigens. Qualitative findings indicated that the intervention influenced parent’s decision to vaccinate their newborn because the barbers were considered a trusted community advisor. Challenges stemmed from the low levels of literacy among community leaders and barbers that resulted in the need for continuous training, low-literacy training materials and supervision.

**Conclusions:**

Efforts to increase vaccine coverage rates in northern Nigeria should consider expanding the role of traditional barbers to encourage parents to accept vaccines.

## Introduction

Immunization coverage rates in Nigeria are among the lowest and most inequitable in the world: the 2018 Nigeria Demographic and Health Survey (NDHS) estimates national coverage of children receiving the third dose of the pentavalent vaccine to be around 50% (ICF 2019). In the northern Nigerian state of Sokoto, only 3% of children receive the third dose of pentavalent vaccine by their first birthday ((UNICEF) 2017). The Routine Immunization (RI) program in Nigeria has faced many challenges including a shortage of vaccines and supplies, poor quality routine data that make planning and delivering adequate services difficult, and insufficient numbers of trained health workers deployed in rural areas (Babalola 2009; Babalola and Lawan 2009; Dunkle et al. 2014; Adeloye et al. 2017; Fatiregun and Etukiren 2014; Ophori et al. 2014). Supply-side issues are frequently cited as the reason children are not fully immunized; however, lack of knowledge and community norms are the primary reasons children are not immunized at all (Babalola 2011). In northern Nigeria, factors at the community level, such as limited knowledge among mothers about the risks associated with not vaccinating their children, misinformation from rumors and distrust of the health system, further exacerbate the system level issues, resulting in few children receiving their first vaccine dose (Michael et al. 2014; Ghinai et al. 2013; Warigon et al. 2016).

Health workers can be effective conduits of information; for example, when mothers receive information during visits to a clinic or are given specially designed immunization cards, they are more likely to vaccinate their children (Brown et al. 2016). A data-driven approach that enabled health workers to identify and remind women to vaccinate their children led to improved immunization coverage during the polio eradication effort (Waisbord et al. 2010). However, the shortage of health workers in Nigeria, particularly in rural areas, can result in limited opportunities for health workers to engage parents about the importance of RI outside the facility and directly in the community (Ophori et al. 2014).

Targeting influential leaders as conduits of information during the polio eradication campaigns has increased the uptake of polio vaccine in poor-performing areas in northern Nigeria (Warigon et al. 2016). Village meetings with community leaders can increase the number of children vaccinated (Owais et al. 2011; Brugha and Kevany 1996). Immunization programs that incorporate mobile reminders are a feasible and acceptable method of reminding women to return to the clinic for vaccinations (Brown et al. 2015). However, communication approaches used in Nigeria rarely enable two-way communication with trusted advisors who can address barriers related to misinformation and misperceptions and build the necessary trust between caregivers and the health system (Oku et al. 2016). Few studies have shown the extent to which community-based volunteers can strengthen demand for RI by improving knowledge and reducing misinformation among parents (Uzondu et al. 2015).

Recognizing that engagement and communication with families and communities are essential, the Sokoto State Primary Health Care Development Agency (SPHCDA) developed a Community Partnership Strategy for RI (SPHCDA 2017). The strategy highlighted the importance of engaging community-based volunteers, such as traditional barbers (known as Wanzams in the predominantly-Hausa speaking communities) to increase community demand and the use of RI services. Traditional barbers are men appointed by the traditional leadership in their communities, and they exist in almost all communities in northern Nigeria. The barbers traditionally shave all newborns’ hair on the seventh day after birth at the newborn’s home as part of the Islamic birth rites. The timing of the traditional barber’s visit provides a unique opportunity to inform parents about the importance of providing birth antigens to their newborns in the first week of life.

In this paper, we evaluate the degree to which newborn identification and referral by traditional barbers affected immunization coverage for birth antigens, as well as examining the challenges and enabling factors experienced with implementing this approach.

## Methods

### Study site

The study team selected 10 Local Government Areas (LGAs) in Sokoto and stratified the LGAs to represent each of the three senatorial zones in the state, with a mix of urban and rural areas and high- and low-performing LGAs based on their reported vaccination coverage rates. LGAs receiving substantial RI program support from partner agencies beyond support sponsored through the partnership with the Bill & Melinda Gates Foundation, the Dangote Foundation and USAID were not included in the sample to minimize bias and contamination (MCSP 2018). LGAs facing security challenges were not included in the sample to protect the safety of data collectors. LGAs in each zone were randomly assigned to intervention and comparison groups.

### Intervention description

**Fig. 1:**
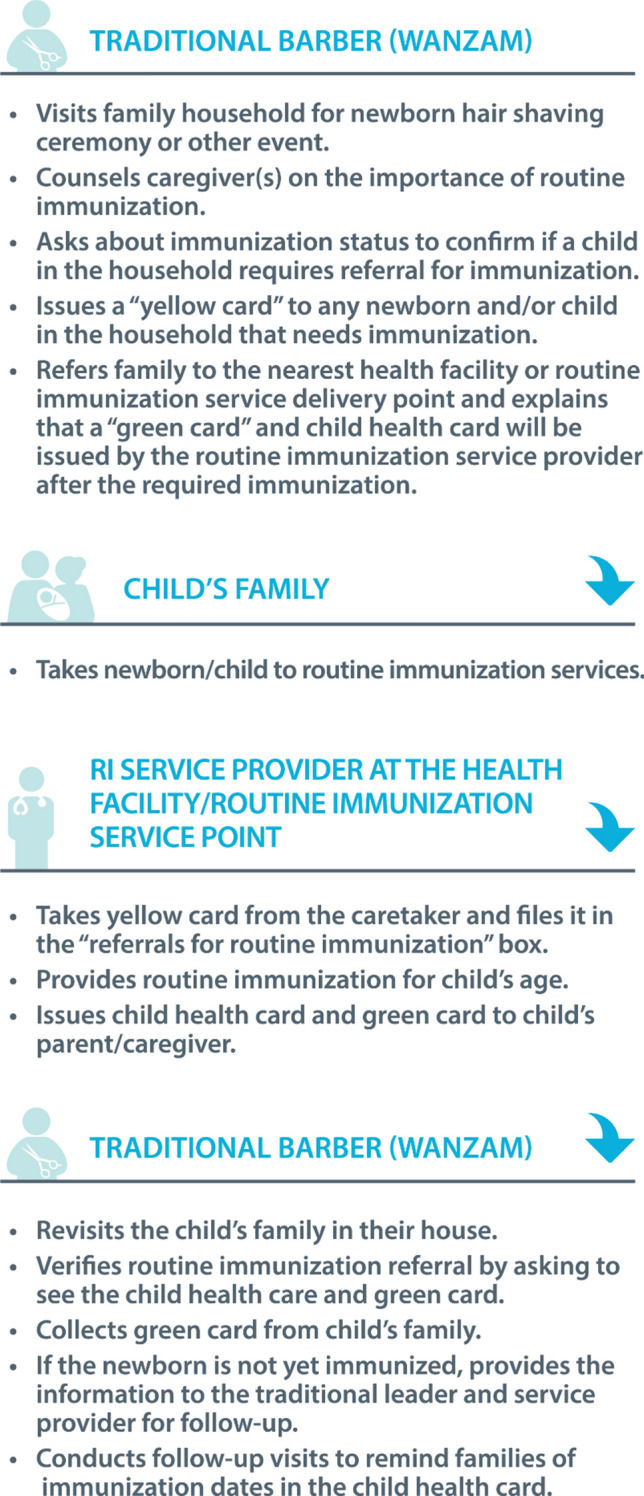
Outline of steps in newborn tracking approach, Nigeria 2017–2018

The Maternal and Child Survival Program (MCSP), funded by the US Agency for International Development (USAID) worked closely with the SPHDCA throughout 2018 to engage with traditional authorities. In northern Nigeria, traditional authorities became an important partner in the Polio Eradication Initiative after the Sultan of Sokoto, a Muslim spiritual leader, publicly announced his support for the initiative (Nasir et al. 2014). The traditional authorities operate within a hierarchical structure from the state down to the community level where they engage directly with community leaders and traditional barbers’ associations. The MCSP supported the SPHCDA in May 2018 to train 1210 traditional barbers who were selected by traditional authorities along with orienting health providers and community leaders in five LGAs in Sokoto to identify and refer newborns for RI; 844 barbers participated in the strategy. The barber’s training included the importance of immunization, how to counsel and educate a family about the benefit of immunization through interpersonal communication and the process of using color-coded referral cards. SPHCDA encouraged traditional barbers to re-visit the family once to confirm compliance with the vaccination referral. However, this visit was not mandated since the traditional barber was not provided with an incentive to repeat visits. There were no follow-up trainings or direct supervision provided to the traditional barbers. However, monthly meetings facilitated by health providers with traditional barbers and community representatives provided an opportunity for questions and feedback and traditional barbers were encouraged to report defaulters to community leaders. SPHCDA program managers also monitored the number of each color card distributed and recovered each month. The steps involved in the approach are described in Fig. 1.

**Fig. 2:**
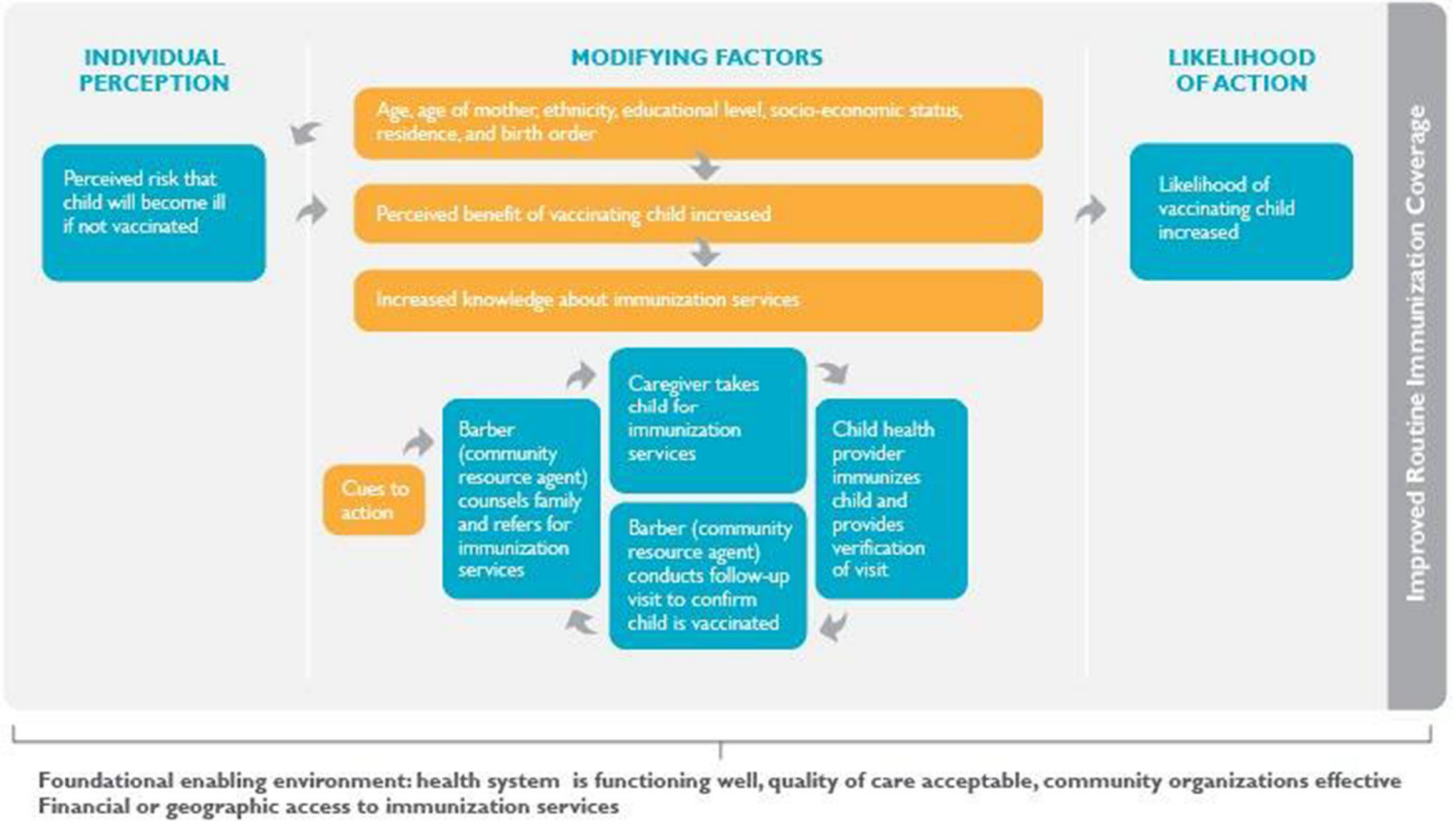
Theory of change for using traditional barbers to identify, and refer newborns for routine immunization services, Nigeria 2017–2018

Figure 2 presents a Theory of Change (ToC) that describes how the strategy was expected to influence immunization coverage. The strategy is grounded in the Health Belief Model, which recognizes that parents will be more likely to vaccinate their child if they understand that the vaccine-preventable illness is serious, that their child is susceptible and that there is an increased threat to their child if they are not immunized (Murele et al. 2014; Rosenstock 1974). The strategy uses the traditional barber to provide information and a cue to action for parents to initiate the use of RI services, grounded in an understanding that social relationships within the community have a strong influence on health behaviors (Ataguba et al. 2016). These relationships can help to mitigate some of the socio-demographic factors that affect immunization coverage (Ataguba et al. 2016; Adenike et al. 2017; Sibeudu et al. 2017).

### Study design

The mixed methods study included quantitative methods to assess differential changes over time in immunization coverage rates associated with the strategy, while explaining how and why changes occurred using qualitative methods.

### Quantitative

The study team administered a pre/post-quasi-experimental survey with the baseline survey implemented before program implementation in December 2017 and the endline survey administered at the conclusion of implementation in October to November 2018.

We used a two-stage stratified sampling procedure. In the first stage, we listed all enumeration areas by ward in each of the 10 selected LGAs. We then used probability proportion to size to select 60 enumeration areas per LGA starting at a random point and then systematically selecting areas using a fixed sampling interval. In total, we sampled 300 enumeration areas for each study group. In the second stage, the study team enumerated all households and screened the household for eligible women. We randomly selected ten households per enumeration area and interviewed 10 women between 15 and 49 years of age who had been married and had a child under the age of 2 years. Individuals under the age of 18 are considered emancipated if they are married or in union. In these instances, verbal consent from the participant was obtained without guardian consent.

We selected women who had a child between 0 and 23 months because this enabled us to follow children who were born during the intervention period and who would have had up to 6 months of exposure to the traditional barber intervention. We estimated a sample size of 1300 children between the ages of 0-5 months per survey area, totaling 2600 children for the two survey areas at baseline and at endline. This sample size was based on a change of 5% points in the proportion of infants 0–5 months receiving BCG at birth in the intervention area, with 80% power to detect a difference, alpha of 0.05 and assumptions of an intracluster correlation of 0.21, determined from DHS data (Lê and Verma 1997) and a 10% non-response rate.

The principal outcomes of the survey were birth antigens including the proportion of infants 0–5 months who received the birth doses of oral polio vaccine (OPV), BCG and hepatitis B (HepB) vaccines. We measured OPV and BCG doses based on data recorded from the child’s vaccination card, and when a vaccination card was not available, the information was based on the mother’s recall. Hepatitis B vaccine was based only on data recorded on the child’s vaccination card. We also collected additional socio-demographic information including household assets (to compute a wealth index following DHS methods (Rutstein and Johnson 2004)), residence, sex of the child and maternal characteristics, such as parity, age and education. We measured changes in knowledge and attitudes related to vaccines. Finally, we assessed exposure to the program by measuring the percent of mothers who received a yellow referral card from a traditional barber during the implementation period.

### Qualitative

The qualitative component of the study included focus group discussions (FGD) and in-depth interviews (IDIs). We conducted interviews at the state level and in a subset of three intervention LGAs (i.e., Dange Shuni, Illela, Shagari) in September 2018, interviewing 18 respondents who were involved in the development and implementation of the strategy. The study team conducted two FGDs with parents who had an infant born in the year preceding the interview (mothers and fathers were interviewed separately), two FGDs with ward development committee members and one FGD with traditional barbers in each of the three LGAs, for a total of 21 FGDs. The study team also conducted four IDIs with village heads and five health providers from each LGA for a total of 27 IDIs at the community level. The study team worked with government and community representatives to purposively select respondents to achieve a geographic mix of individuals involved in or exposed to the program’s intervention. Moderators conducted the interviews with community members in the local language of Hausa. Interviews were recorded using a digital audio recorder and translated and transcribed into English.

### Analysis

To measure the effect of the program, we estimated simple logistic regression models on each birth antigen while controlling for demographic characteristics using Stata SE 15. Variables were selected for inclusion in an adjusted logistic regression model if they satisfied the criterion of *p* < 0.05 in the simple logistic regression model. To assess the effect of the program, we controlled for the study period (baseline and endline) and study group. We also included a measure to assess whether the respondent had received a yellow referral card from a traditional barber to measure the effect of program exposure. We performed diagnostic tests to check for specification errors, goodness of fit and collinearity with the regression models. We accounted for the design effect by assigning the enumeration areas as the primary sampling unit for analysis.

We used thematic analysis techniques to identify themes emerging from the qualitative data. We considered how traditional barbers influence caregiver decisions to seek immunization services and explored the challenges and opportunities associated with engaging traditional barbers to refer newborns for immunization services. We used the ToC model to develop codes related to how the intervention influenced the outcomes such as parental knowledge and identified emerging themes to code challenges (e.g., the need for incentives) and enabling factors (e.g., traditional leader support). We applied the codes in Nvivo 12. Findings were described and compared across subgroups. We used findings from the qualitative data to contextualize the quantitative data to develop a more comprehensive understanding of how the program was implemented and what factors related to the context and implementation influenced the results.

## Results

Table 1 presents the characteristics of the study participants by study arm at baseline. The groups were similar in residence, mother’s age, parity and visitation by a traditional barber after birth. Women in the intervention areas tended to have less education and reside in wealthier households.

**Table 1:** Background characteristics of mothers with child between 0 and 5 months at baseline, Nigeria, 2017

	Baseline comparison (%)	Baseline intervention (%)	*P*-value
Urban residence	7.8	7.9	0.962
*Wealth status*			
Poor	35.0	28.7	0.063
Middle	35.8	34.9	
Rich	29.2	36.4	
*Mother’s age group*			
15–19	13.5	15.6	0.337
20–34	67.3	67.9	
35–49	19.1	16.5	
*Mother’s education*			0.000
No education	85.0	91.5	
Primary	8.5	5.9	
Secondary+	6.5	2.6	
*Parity*			
1 child	12.7	14.1	0.579
2-4 children	46.9	48.1	
5+	40.4	37.9	
Male child	45.9	49.5	0.193
Visited by traditional barber after birth	87.2	85.9	0.595
*N*=	695	697	

Findings from the qualitative component of the study sought to explain how traditional barbers influenced parent’s decision to seek immunization services. Parents trusted traditional barbers and found that the messages were shared consistently throughout the community and reinforced their beliefs that the advice benefited a child’s health.

*Before he comes with the explanation, I already saw him explaining to others, although he did explain to me, too, and I trusted him; his methods are good, and his advice are good because a child requires good health to be strong and active. (Shagari Father, Sh16)*

Both parents and traditional barbers commented on the importance of dialogue when discussing immunizations. The two-way communication enables parents to ask questions and gives barbers more time to address barriers that prevent parents from seeking vaccination services. In addition, because traditional barbers initiate conversations following traditional ceremonies, the barber can address both parents. Since mothers often must seek permission from the husband before taking the child to the health facility, the barber's contact with the father encourages his support as well.

*Our work is giving the cards as referrals to women but we do more talking about letting them understand the importance of immunization rather than just giving and collecting back cards; we take about 30 minutes or more trying to convince women or their husbands to make sure they voluntarily accept doing it or at least to let them visit the facility to get more health talk from health workers. Then we offer the yellow card after the long talk and after some days we re-visit the household and ask if they really went to the facility. (Dange Traditional Barber, D1)*

The linkages between the traditional community leaders and the barbers provide an added layer of support. Traditional barbers are already a part of the traditional structures and are encouraged to report parents who refuse to vaccinate their children directly to community leaders for follow-up.

*Whenever there is a newborn, the traditional barber will counsel the father and issue him a card to take to the health facility, and in case the father refuses, the barber shouldn’t fight him but rather report him to the community head, to address his issues. (Dange WDC member, D3)*

We examined changes in the pathways to improved immunization coverage described in the ToC. Table 2 shows that 16.6% of the intervention group had received a yellow card from the traditional barber at endline. We found at baseline that approximately 18% of women in both the intervention and the comparison groups had knowledge about the need to immunize a newborn within the first week of life. At endline, we found that this increased to 39% in the intervention group (a 21% point increase) compared to 27% in the comparison group (a 9% point increase). Similarly, when asked if the mother knew the child should be vaccinated five times, we found an increase of 10.9% to 18.5% in the intervention group (7.6% points) compared to an increase of 11.8% to only 14.2% (2.4% points) in the comparison group. The SPHCDA asked the traditional barbers to inform the parents about these two key messages during their visits. We also asked mothers if they believed that vaccinations conferred a benefit to the child. In the intervention groups, we found a nearly 9% point increase in the number of mothers who believed that their child was more likely to get sick if they had not been vaccinated; this belief did not change between baseline and endline among mothers in the comparison group. We also found a larger increase in mothers who said they would take their child for vaccination services in the intervention group. We measured changes in immunization coverage of the three birth antigens and found that the increase in coverage was the greatest among the intervention group for all three antigens

**Table 2:** Key indicators according to the theory of change as reported by mothers with child between 0 and 5 months at baseline and endline, Nigeria, 2017–2018

	Baseline Comparison (%)	Endline Comparison (%)	Baseline intervention (%)	Endline Intervention (%)
*Cue to Action*				
Mother receives an yellow card from traditional barber	0.0	1.5	0.0	16.6
*Knowledge of immunization*				
Mother knows newborn should be immunized within the first week after birth	18.3	27.2	18.7	39.3
Mother knows a child should be brought for vaccination five times	11.8	14.2	10.9	18.5
*Benefit of vaccination*				
Mother believes if children are not vaccinated they are more likely to get sick	59.6	59.8	51.4	59.9
*Likelihood of vaccinating child*				
Mother says she will definitely take the child for vaccination	64.8	67.6	50.4	64.3
*Immunization coverage of birth antigens*				
Hepatitis B (card only)	10.8	16.6	6.7	15.0
BCG (card and recall)	20.3	27.7	15.8	27.8
Polio (card and recall)	21.6	25.6	17.2	25.4
*N*=	695	697	697	633

We estimated logistic regressions to determine the effect of the traditional barber intervention on the three birth antigens (BCG, OPV and HepB). We first conducted an intention to treat analysis by computing an interaction variable (study period by study group) to assess whether the intervention group was significantly more likely to achieve higher coverage of the three birth antigens. We did not find a significant effect by study group over time. However, this may be due to the short implementation period, which limited the program’s opportunity to reach its full effect. We then estimated a second adjusted model where our key predictor was whether the mother had received a yellow card from a traditional barber to determine if exposure to the intervention influenced birth antigen coverage. We controlled for maternal age, education, and wealth status (polio only). Table 3 presents the odds ratios and confidence intervals for both the unadjusted and adjusted logistic regression models for each study outcome. We found that among mothers who received a yellow referral card from a traditional barber, infants 0-5 months of age were two to three times more likely to have received their birth antigens. (BCG (AOR = 3.0, *p* < 0.0001), OPV0 (AOR = 2.4, *p* < 0.0001), and HepB (AOR = 2.7, *p* < 0.0001).

**Table 3:** Odds ratio and confidence intervals of demographic characteristics and effect of intervention by birth antigen outcomes (as reported by card and mother’s recall) among children 0–5 months, Nigeria, 2017–2018

	BCG unadjusted	BCG adjusted	Polio0 unadjusted	Polio0 adjusted	Hepatitis B0 unadjusted	Hepatitis B0 adjusted
Urban residence	0.8 [0.5–1.2]	na	0.9 [0.6–1.4]	na	0.9 [0.6–1.4]	na
*Wealth status (reference: poor)*						
Middle	0.8 [0.7–1.1]	na	0.7 [0.6–0.9]**	0.7 [0.6–0.9]*	0.8 [0.6–1.0]	na
Rich	1.1 [0.9–1.5]	na	1.0 [0.7–1.3]	0.9 [0.7–1.2]	1.1 [0.8–1.5]	na
*Mother’s age group (reference: 15*–*19)*						
20–34	1.6 [1.2–2.2]**	1.6 [1.2–2.3]**	1.3 [0.9–1.8]	1.3 [0.9–1.8]	1.9 [1.2–2.9]**	1.9 [1.2–3.0]**
35–49	1.6 [1.1–2.3]*	1.6 [1.1–2.3}*	1.3 [0.9–1.9]	1.3 [0.9–1.8]	1.4 [0.9–2.2]	1.4 [0.9–2.3]
*Mother’s education (reference: no education)*						
Primary	1.5 [1.0–2.2]*	1.5 [1.0–2.2]*	1.2 [0.8–1.7]	1.2 [0.8–1.7]	1.4 [0.9–2.1]	1.3 [0.8–2.1]
Secondary +	2.0 [1.3–3.0]**	2.0 [1.3–3.2]**	1.6 [1.1–2.5]**	1.6 [1.0–2.6]*	2.3 [1.4–3.7]**	2.2 [1.4–3.7]**
*Parity (reference: 1 child)*						
2–4 children	1.2 [0.9–1.6]	na	1.1 [0.8–1.5]	na	1.3 [0.9–1.9]	na
5+	1.2 [0.9–1.6]	na	1.3 [0.9–1.1]	na	1.3 [0.8–1.9]	na
Male child	1.1 [0.9–1.3]	na	1.1 [0.9–1.3]	na	1.0 [0.8–1.2]	na
Group: intervention (reference: comparison)	0.9 [0.7–1.1]	0.8 [0.5–1.1]	0.9 [0.7–1.1]	0.8 [0.6–1.1]	0.8 [0.5–1.1]	0.7 [0.4–1.0]
Time: endline (reference: baseline)	1.7 [1.4–2.1]***	1.5 [1.2–2.0]**	1.4 [1.2–1.7]**	1.2 [1.0–1.6]	2.0 [1.5–2.5]***	1.7 [1.2–2.3]**
Interaction (time*group)		1.1 [0.7–1.6]		1.1 [0.7–1.7]		1.1 [0.6–2.1]
Received referral card from barber	3.4 [2.2–5.2]**	3.0 [1.9–4.9]***	2.5 [1.7–3.7]***	2.4 [1.6–3.6]***	3.0 [1.9–4.6]***	2.7 [1.6–4.4]***
*N*=	2639	2639	2639	2639	2639	2639

*na* not applicable

*p  = < 0.05; **p  = < 0.01; ***p  < 0.001

The design of the traditional barber intervention leveraged several contextual factors that helped to strengthen the overall program. First, the intervention worked with traditional structures. In northern Nigeria, the traditional barbers have an association or union that acts as a network of community-based resource persons deployed throughout the state. This facilitated the selection of traditional barbers to participate in the intervention.

*The barbers have a structured union with representatives in the state, LGAs, wards and community levels. The Sultanate or Emirate Council instructed the traditional barber’s cabinet to nominate barbers who were be engaged in the program through their hierarchy down to community level. (State Advocacy, Communication and Mobilization, S2)*

The intervention was designed to create linkages between the traditional barbers and the community leaders and health providers. These linkages allowed traditional barbers to receive support from community leaders and health providers during periodic meetings that helped to provide continuous feedback throughout implementation.

*We had the meeting in this facility in the presence of the officer in charge. The meeting we had with the community leaders, they reminded us of the importance of giving this referral card and making sure the woman takes the card to the health facility. (Illela, Traditional barbers, I1)*

Respondents identified several challenges in implementing the traditional barber approach. First, periodic monitoring on the progress of the approach determined that some traditional leaders selected friends and family members to act as traditional barbers to receive training per diems leaving their villages without a trained traditional barber. Second, the SPHCDA trained traditional barbers, health providers and community leaders on the key elements of the intervention. Although the training was adapted to support the low levels of literacy among traditional barbers, government stakeholders noted that continuous training would be needed to ensure proper understanding of the approach. Specifically, government officials noted that some traditional barbers were confused with the cards and distributed them incorrectly.

*Here, I can tell you there are a lot of issues, because some of the traditional barbers still do not understand the work. Some of them were thinking that the referral card should be given to the parents only if the baby is born ill or got sick just after delivery. But during sensitization visit on Friday, we corrected that mistake and gave them the right information; In the light of this, we suggest that the traditional barbers be retrained again and again; the training should be done for both the barbers and the RI service providers because they need to understand how each of them work and the role they play. (Shagari, LIO, Sh5)*

Third, several government officials, community leaders and traditional barbers also mentioned that they felt traditional barbers should receive an incentive for their efforts. Although families pay traditional barbers to perform traditional ceremonies, there is no incentive for the traditional barber to return a week later to verify that the parents have taken their child for vaccination. In situations where the traditional barber does not have a financial reason to return to the community, this can result in some barbers not completing the follow-up visit.

*Distance is a challenge; sometimes it is difficult for the traditional barber to go back for follow*-*up after issuing the yellow referral card because of distance. He goes for the naming ceremony because he will benefit in one way or another by attending; but for follow*-*up, he may not find it easy to return, especially if the place is very far, unless there is another naming there. (State Advocacy, Communication and Mobilization, S2)*

Supervision meetings also found that some trained traditional barbers did not participate in the program after receiving training because they had anticipated but had not received a financial benefit for their effort. Finally, in some cases, parents were resistant to traditional barbers’ messages and refused to take their newborns for immunizations after exposure to the program.

## Discussion

Our findings describe the opportunities and challenges in engaging traditional barbers in northern Nigeria to strengthen the demand for RI services at the community level. We found that mothers who received a yellow referral card from a traditional barber were significantly more likely to vaccinate their children with birth antigens. This indicates that the approach when applied as designed was effective at improving vaccination coverage. However, we found that the exposure to the intervention was limited to 16% of mothers of infants interviewed indicating that implementation challenges prevented the approach from fully reaching the target population thereby mitigating the full impact.

The program sought to engage directly with parents through two-way communications. The traditional barber–a trusted community agent–was able to address parent concerns and provide information about where to obtain services as well as the number of doses and timing of vaccinations. The literature suggests that taking this type of approach within the context of a broader multi-component behavior change strategy could lead to a greater effect on outcomes (Jarrett et al. 2015). Another important consideration is that the traditional barber approach engaged parents during a traditional ceremony, which allowed barbers to speak directly with fathers who, in the culture of northern Nigeria, make decisions about when a mother can take a child for care. Given that previous studies have found that women who lack decision making autonomy have a lower likelihood of immunizing their children, the results of the traditional barber intervention should be considered for future immunization programming in this region (Antai 2012).

State and LGA level program managers worked effectively to build linkages with traditional authorities and existing structures during implementation which enabled the program to reach communities over a short period. These connections leveraged the existing role of traditional barbers and expanded their responsibilities to include vaccination referrals which increased their social status within their communities and served as a source of motivation to sustain the intervention. Periodic meetings allowed traditional barbers to receive feedback and support from traditional leaders and health providers, which improved their ability to engage with parents. Connections between traditional barbers and community leaders also afforded traditional barbers the opportunity to refer parents who refused services to a community leader to reinforce messages about the importance of immunization. Our findings support previous studies that found involving religious or traditional leaders particularly in populations with low immunization coverage rates can have a positive effect on outcomes (Nasir et al. 2014; Nwaze and Mohammed 2013). Future programs in Nigeria should consider integrating traditional and religious leaders to improve the results of communication efforts at the community level.

While the traditional barber approach was successful in increasing birth antigen coverage rates in our study, several challenges hindered implementation. Some leaders of traditional barbers selected friends and family members or barbers who were not favored by the community to participate in the program. As a result, some communities were left without a trained traditional barber to provide the referral cards at the time of services. Future programs should consider engaging with community members through a participatory process to ensure that traditional barbers selected are those that are most frequently consulted by the community (Beran 2018).

Many of the traditional barbers selected for the program were illiterate and could not benefit from supplemental written materials to support their communication with parents following the initial training. As a result, messages from the traditional barbers were not always transmitted accurately to parents and the short implementation period did not allow for follow-up trainings to correct knowledge gaps. Periodic refresher trainings should be considered going forward to ensure that traditional barbers are able to accurately transmit messages on the immunization schedule and antigens.

Some traditional barbers anticipated a financial benefit for participating in the program and lost motivation when asked to provide services without compensation. This occurred particularly when they were asked to return to remote communities without reimbursement for their time or transport. Previous studies have found that to ensure retention of community agents like the traditional barbers, they require continuous training, supervision and monetary and or non-monetary incentives (Ludwick et al. 2014; Kasteng et al. 2016). While the program encouraged the barbers to re-visit the family to determine if they had complied and received the green card from the health providers, this did not always happen. As a result, it may be more effective to incorporate this second visit when the barber makes a traditional visit for the celebration at the end of the postpartum confinement period which occurs 40 days after birth. Future programs should also ensure that barbers are informed about their voluntary participation while working closely with health agencies and traditional leaders

Finally, the full impact of this intervention with traditional barbers in northern Nigeria may have been greater with a longer implementation period and greater exposure to the approach; MCSP’s work in Nigeria ended before the study was able to reach a full 12-month birth cohort and only 16% of respondents reported receiving a referral card from a traditional barber.

### Conclusion

The newborn identification and referral approach demonstrated that when mothers are provided with referral cards by traditional barbers, they are more likely to access vaccination services. The approach offers important lessons on how community resource partners such as traditional barbers can be leveraged to increase demand for vaccination services and address vaccine hesitancy particularly in a setting that has encountered resistance due to misinformation and rumors. Further research will be required to understand how implementation can be strengthened to reach a broader segment of the target population.

## Data Availability

All data relevant to this publication can be obtained by request to the authors.
